# Decreased nitrite reductase activity of deoxyhemoglobin correlates with platelet activation in hemoglobin E/ß-thalassemia subjects

**DOI:** 10.1371/journal.pone.0203955

**Published:** 2018-09-20

**Authors:** Attaphon Chamchoi, Sirada Srihirun, Kittiphong Paiboonsukwong, Thanaporn Sriwantana, Piyadon Sathavorasmith, Kovit Pattanapanyasat, Rhoda Elison Hirsch, Alan N. Schechter, Nathawut Sibmooh

**Affiliations:** 1 Molecular Medicine Graduate Program, Multidisciplinary Unit, Faculty of Science, Mahidol University, Bangkok, Thailand; 2 Department of Pharmacology, Faculty of Dentistry, Mahidol University, Bangkok, Thailand; 3 Thalassemia Research Center, Institute of Molecular Biosciences, Mahidol University, Nakhon Pathom, Thailand; 4 Department of Pharmacology, Faculty of Science, Mahidol University, Bangkok, Thailand; 5 Center of Excellence for Flow Cytometry, Department of Research and Development, Faculty of Medicine Siriraj Hospital, Mahidol University, Bangkok, Thailand; 6 Departments of Medicine (Hematology), Albert Einstein College of Medicine, Bronx, New York, New York, United States of America; 7 Department of Anatomy and Structural Biology, Albert Einstein College of Medicine, Bronx, New York, New York, United States of America; 8 Molecular Medicine Branch, National Institute of Diabetes and Digestive and Kidney Diseases, National Institutes of Health, Bethesda, Maryland, United States of America; Boston University, UNITED STATES

## Abstract

Nitric oxide (NO) can be generated from nitrite by reductase activity of deoxygenated hemoglobin (deoxyHb) apparently to facilitate tissue perfusion under hypoxic condition. Although hemoglobin E (HbE) solutions have been shown to exhibit decreased rate of nitrite reduction to NO, this observation has never been reported in erythrocytes from subjects with hemoglobin E/ß-thalassemia (HbE/ß-thal). In this study, we investigated the nitrite reductase activity of deoxyHb dialysates from 58 non-splenectomized and 23 splenectomized HbE/ß-thal subjects compared to 47 age- and sex-matched normal subjects, and examined its correlation with platelet activity. Iron-nitrosyl-hemoglobin (HbNO) was measured by tri-iodide reductive chemiluminescence as a marker of NO generation. HbNO produced from the reaction of nitrite with deoxyHb dialysate from both non-splenectomized and splenectomized HbE/ß-thal subjects was lower than that of normal (AA) hemoglobin subjects. P-selectin expression, a marker of platelet activation, at baseline and in reactivity to stimulation by adenosine diphosphate (ADP), were higher in HbE/ß-thal subjects than normal subjects. HbNO formation from the reactions of nitrite and deoxyHb inversely correlated with baseline platelet P-selectin expression, HbE levels, and tricuspid regurgitant velocity (TRV). Nitrite plus deoxygenated erythrocytes from HbE/ß-thal subjects had a lower ability to inhibit ADP-induced P-selectin expression on platelets than erythrocytes from normal subjects. We conclude that deoxyHb in erythrocytes from HbE/ß-thal subjects has a decreased ability to reduce nitrite to NO, which is correlated with increased platelet activity in these individuals.

## Introduction

Nitrite anion (NO_2_^-^), present in the circulation, is a bioactive source of NO. Nitrite represents a storage form of NO as it is more stable and can be reduced to NO via nitrite reductase activity of deoxyHb to facilitate blood flow under hypoxia [1–4]. Nitrite is contained in erythrocytes and a variety of tissues [5]. Under hypoxic conditions in which NO production by endothelial nitric oxide synthase is compromised, the bioactivity of nitrite is achieved following its reduction to NO when hemoglobin oxygen saturation decreases [6,7]. This provides bioactive NO required for vasodilation, platelet inhibition, and promotion of oxygen supply to tissues. Impaired NO bioavailability owing to endothelial dysfunction is associated with metabolic and cardiovascular disorders such as hypercholesterolemia, hypertension, diabetes mellitus, and smoking [8].

Endothelial dysfunction is present in thalassemia as a consequence of multiple factors, including oxidative stress, increased cell-free hemoglobin, and chronic hypoxia. Decreased nitrite levels in blood were reported in HbE/ß-thal children, which were associated with disease severity, cell-free hemoglobin, and lipid peroxidation [9]. Transfusion of packed erythrocytes resulted in an increase in erythrocytic nitrite. Reduction in flow-mediated dilation of the brachial artery in response to reactive hyperemia was also reported in HbE/ß-thal patients [10,11]. Plasma NO metabolites and prostaglandin E_2_ decreased while soluble thrombomodulin (a marker of endothelial activation/injury) increased in HbE/ß-thal patients [11]. The endothelial dysfunction with decreased NO contributes to vascular complications in thalassemia, including pulmonary hypertension, platelet hyperactivity, and thromboembolism [12].

Apart from endothelial dysfunction, it has been proposed that a decrease in NO availability as a result of reduced nitrite reductase activity of deoxyHbE may give rise to a diverse clinical spectrum in HbE/ß-thal [13,14] such as variations in anemia, transfusion requirements, and occurrence of cardiovascular disorders. As deoxygenated hemoglobin in erythrocytes can catalyze nitrite reduction to NO resulting in platelet inhibition [15], it is possible that the reduced rate of HbE-mediated production of NO may be a factor in aggravating platelet activation, a key event leading to thrombosis and vascular complications in HbE/ß-thalassemia.

Here, we hypothesized that deoxyHb of HbE/ß-thal subjects would have a decreased ability to reduce nitrite to NO, resulting in a decrease in NO availability and increase in platelet activation. To examine the impact of splenectomy on platelet activity and vascular dysfunction [16,17], both non-splenectomized and splenectomized HbE/ß-thal subjects were recruited in this investigation. NO produced from the reaction between nitrite and deoxyHb from healthy and HbE/ß-thal subjects was determined as HbNO. Based on the report that platelet P-selectin expression was elevated in HbE/ß-thal patients and correlated with TRV [18], P-selectin expression and TRV were used as markers of platelet activation and estimated pulmonary artery pressure, respectively. Correlations of HbNO formation with P-selectin expression on platelets, HbE levels, and TRV were analyzed. Furthermore, the *in vitro* platelet inhibition by nitrite in the presence of deoxygenated erythrocytes from healthy and HbE/ß-thal subjects was examined.

## Materials and methods

### Subjects

This study was approved by the Ramathibodi Hospital Ethics Committee (ID12-56-13). Written informed consent was obtained from all subjects in accordance with the Declaration of Helsinki. Forty-seven normal (AA) hemoglobin subjects, 58 non-splenectomized, and 23 splenectomized HbE/ß-thal subjects were enrolled in this study. Hemoglobin types of normal and HbE/ß-thal subjects were checked by high performance liquid chromatography at the Thalassemia Research Center, Institute of Molecular Biosciences, Mahidol University. Clinical blood tests were determined at Pathological Laboratory of Faculty of Medicine Ramathibodi Hospital. As splenectomy is a risk factor of pulmonary hypertension [19], measurement of TRV in splenectomized HbE/ß-thal subjects by echocardiography was performed at Cardiology Unit, Department of Medicine, Faculty of Medicine Ramathibodi Hospital. To avoid the effect of transfusion of erythrocytes on platelet activation [20], the HbE/ß-thal subjects did not receive a transfusion for at least 2 weeks prior to enrollment.

### Reagents

Mercury (II) chloride (HgCl_2_) and 2-(4-carboxyphenyl)-4,4,5,5-tetramethylimidazole-1-oxyl 3-oxide (C-PTIO) were purchased from Merck (Darmstadt, Germany). Monoclonal antibodies: FITC-labeled anti-CD42a, PE-labeled anti-CD62P, FITC-labeled anti-mouse IgG1, and PE-labeled anti-IgG1 were purchased from BD Biosciences (San Jose, CA). Other chemicals were purchased from Sigma Aldrich (St. Louis, MO).

### Blood sample collection

Fasting venous blood was obtained by venipuncture and aliquoted into tubes containing 3.8% sodium citrate (9:1) for the platelet study and K_2_EDTA (1.8 mg/ml) for the study of the reaction between nitrite and deoxyHb.

### Preparation of hemoglobin dialysate

Fresh blood was collected using K_2_EDTA as an anticoagulant, and centrifuged at 2,000×*g* for 5 min at 4 °C to separate erythrocytes. The obtained packed erythrocytes were washed thrice with 10 mM phosphate buffered saline (PBS), pH 7.4 and lysed with 3 volumes of distilled water to make the hemolysate. In order to isolate hemoglobin, neutralized saturated ammonium sulfate solution was added into hemolysate to obtain 20% saturation [21], left on ice for 15 min and centrifuged at 20,000×*g* for 1 h at 4 °C. The red clear supernatant was collected and dialyzed against deionized water at 4 °C with gentle stirring for 48 to 72 h. The dialysate was collected, and the hemoglobin in the dialysate was measured by commercial Drabkin’s reagent and kept at -80 °C. At the time of analysis, the dialysate was thawed at 4 °C and diluted with 10 mM PBS pH 7.4 to desired concentration.

### Measurement of blood nitrite

Heparinized whole blood (143 units/10 mL) was mixed with a nitrite-preserving solution (0.8 M potassium ferricyanide, 10 mM N-ethylmaleimide and 1% NP-40 in a 5:1 dilution) immediately after blood drawing [22], and kept frozen at -80 °C. Nitrite in whole blood was measured by triiodide-based chemiluminescence [23] using a chemiluminescence NO detector (Eco Medics Analyzer CLD88, Duernten, Switzerland).

### Measurement of NO production from nitrite plus deoxyHb

The reaction of nitrite with deoxyHb dialysates in PBS pH 7.4 at 37 °C was studied in sealed cuvettes. Hemoglobin dialysates (50 μM heme) from normal or HbE/ß-thal subjects were introduced into cuvettes and continuously purged with helium gas for 3 min prior to addition of 0.5 μM sodium nitrite. This procedure increased deoxyHb from 14.3 to 91.0% as measured by spectral deconvolution. HbNO was measured as a marker of NO formation by sequential chemical reactions [23]. Briefly, the reaction was stopped by the addition of 5% acid sulfanilamide solution (9:1) and incubated for 5 min. Then, the samples were incubated with 50 mM HgCl_2_ (9:1) for 5 min, and with 5% acid sulfanilamide solution (9:1) for another 5 min. The amounts of HbNO in the final reaction mixture were measured by triiodide-based chemiluminescence [23]. Heme was measured by Drabkin’s reagent. HbNO was expressed as nmol NO per heme.

### Measurement of platelet activation by flow cytometry

Citrated whole blood was diluted with PBS pH 7.4 (1:10). Fifty microliters of the diluted whole blood were dispensed in a 12×75-mm polystyrene tube and incubated with 5 μL of fluorescein isothiocyanate (FITC)-labeled anti-CD42a (platelet glycoprotein IX) and phycoerythrin (PE)-labeled anti-CD62P (P-selectin). FITC-labeled anti-mouse IgG1 and PE-labeled anti-IgG1 were used as isotype controls for FITC-labeled anti-CD42a and PE-labeled anti-CD62P, respectively [18,24]. The incubation was done at room temperature in the dark for 15 min. The fluorescence-labeled samples were resuspended in 500 μL of PBS and analyzed immediately for baseline P-selectin expression using a FACS Calibur (BD Biosciences, San Jose, CA).

During the platelet granule release reaction, large amounts of ADP are released which is important in mediating platelet aggregation, causing platelets to adhere to one another [25]. ADP-induced platelet P-selectin expression was used as a marker of platelet activity as it was reported to be increased in HbE/ß-thal and related with vascular complication such as pulmonary artery pressure [18]. The aliquot of fluorescence-labeled samples were further incubated with 1 μM ADP at room temperature for 15 min. Then, 10 μL of samples were fixed with 500 μL of 1% paraformaldehyde at 4 °C for 15 min and analyzed for ADP-induced P-selectin expression using a FACS Calibur (BD Biosciences, San Jose, CA).

### *In vitro* platelet inhibition by nitrite plus deoxygenated erythrocytes

Platelet-rich plasma (PRP) and deoxygenated erythrocytes were prepared as described previously [15]. Citrated whole blood samples from normal subjects were centrifuged at 120×*g* for 10 min. PRP on the upper layer was gently aspirated into a new conical tube. Then, the stained PRP samples were prepared by diluting PRP with deoxygenated 10 mM PBS pH 7.4 on a 1:4 (vol/vol) ratio, and the FITC-labeled anti-CD42a and PE-labeled anti-CD62P were added into diluted PRP suspensions at 1:50 (vol/vol) ratio. The packed erythrocytes from centrifuged whole blood samples were gently collected and washed thrice with 5× volumes of 10 mM PBS pH 7.4, and resuspended in 10 mM PBS pH 7.4 to obtain erythrocytes in suspension normalized to a 50% hematocrit. Deoxygenated erythrocytes were prepared by blowing helium gas above the suspension with gentle rotation at room temperature for 30 min in a sealed flask. The control untreated erythrocytes had partial pressure of oxygen (PO_2_) 56.0 mmHg and oxygen saturation (SO_2_) 90.3%, while deoxygenated erythrocytes had PO_2_ 20.2 mmHg and SO_2_ 59.1%.

Stained PRP samples were transferred into sealed silicone-coated glass cuvettes and equilibrated in a 37 °C water bath for 5 min. Then, the stained PRP were pre-incubated for 15 min with or without 20 μM ODQ (1H-[1,2,4]oxadiazolo[4,3-a]quinoxalin-1-one, a soluble guanylate cyclase inhibitor) or 200 μM C-PTIO (2-(4-carboxyphenyl)-4,4,5,5-tetramethylimidazole-1-oxyl 3-oxide, an NO scavenger) [15]. Afterwards, the erythrocytes from healthy or HbE/ß-thal subjects were added into cuvettes to obtain a 20% hematocrit, and incubated with 0.5 μM sodium nitrite at 37°C for 10 min. Then, 20 μM ADP was added into each tube and incubated further for 10 min. The ADP-induced platelet activation was stopped by fixing 10 μL of samples with 500 μL of 1% paraformaldehyde at 4 °C for 15 min. Platelet P-selectin expression was determined by FACS Calibur (BD Biosciences, San Jose, CA).

### Statistical analysis

Statistical analysis was performed using Predictive Analysis Software (PASW) Statistics version 18.0 (IBM Corp., Armonk, New York, USA). The statistical significance of the data was determined by parametric (unpaired *t*-test or ANOVA) or nonparametric methods (Mann-Whitney U test or Kruskal-Wallis test), depending on normality as indicated. Data were expressed as mean ± SD or median with interquartile range. *P* values < 0.05 were considered statistically significant.

## Results

### Subjects’ characteristics

Forty-seven normal (AA) hemoglobin subjects, 58 non-splenectomized HbE/ß-thal subjects, and 23 splenectomized HbE/ß-thal subjects were enrolled in this study (Table 1). Subjects in non-splenectomized and splenectomized HbE/ß-thal groups had lower hemoglobin and hematocrit than normal subjects. Splenectomized HbE/ß-thal subjects had higher platelet count than normal and non-splenectomized HbE/ß-thal subjects. Blood nitrite levels were higher in HbE/ß-thal than normal subjects. Both non-splenectomized and splenectomized HbE/ß-thal subjects had higher blood nitrite levels than healthy subjects. TRV (mean ± SD: 2.87 ± 0.54 m/s) was measured in splenectomized HbE/ß-thal subjects who had risks or clinical features of pulmonary hypertension.

**Table 1:** Demographic and laboratory data.

	Normal	Hemoglobin E/ß-thalassemia	
		Non-splenectomized	Splenectomized
n	47	58	23
Age, years	27 (25–37)	33 (25–39)	25 (22–41)
Sex, male/female	21/26	27/31	10/13
Hemoglobin, g/dL	13.3 ± 1.5	7.6 ± 1.4^a^	7.5 ± 1.4^a^
Hematocrit, %	40.3 ± 4.3	24.0 ± 5.7^a^	22.2 ± 3.5^a^
White blood cell count, ×10^9^/L	6.9 ± 1.5	6.8 ± 1.5	11.0 ± 4.8^a^^,^ ^b^
Platelet count, ×10^3^/μL	254.7 ± 60.0	199.9 ± 92.0	612.2 ± 182.9^a^^,^ ^b^
Aspartate aminotransferase, U/L	22.1 ± 9.0	40.5 ± 20.1^a^	58.1 ± 29.6^a^^,^ ^b^
Blood urea nitrogen, mg/dL	10.5 ± 3.7	12.2 ± 4.1	12.1 ± 5.2
Creatinine, mg/dL	0.8 ± 0.2	0.6 ± 0.2^a^	0.5 ± 0.3^a^
Ferritin, ng/mL	-	633.1 (336.8–1,552.0)	1,428.0 (606.5–2,575.0)^d^
Indirect bilirubin, mg/dL	0.4 (0.2–0.5)	2.3 (1.3–3.9)^c^	3.5 (2.5–3.9)^c^
Duration of splenectomy, years	-	-	19 (16–28)
Blood transfusion, %	-	27.6 (16/58)	82.6 (19/23)
Duration from last transfusion, days*	-	150 (51–365)	30 (21–60)^d^
Blood nitrite, nM	163.6 ±40.9	233.2±95.5^a^	217.8±95.8^a^

Values represent the mean ± SD or median (interquartile range) as appropriate.

*Data of transfusion-dependent subjects.

^a.^ *P* < 0.05 compared with normal subjects (ANOVA with Bonferroni post hoc test).

^b.^ *P* < 0.05 compared with non-splenectomized HbE/ß-thal subjects (ANOVA with Bonferroni post hoc test).

^c.^ *P* < 0.05 compared with normal subjects (Kruskal-Wallis test).

^d.^ *P* < 0.05 compared with non-splenectomized HbE/ß-thal subjects (Mann-Whitney U test).

### Nitrite reduction to NO by deoxyHb

**Fig 1:**
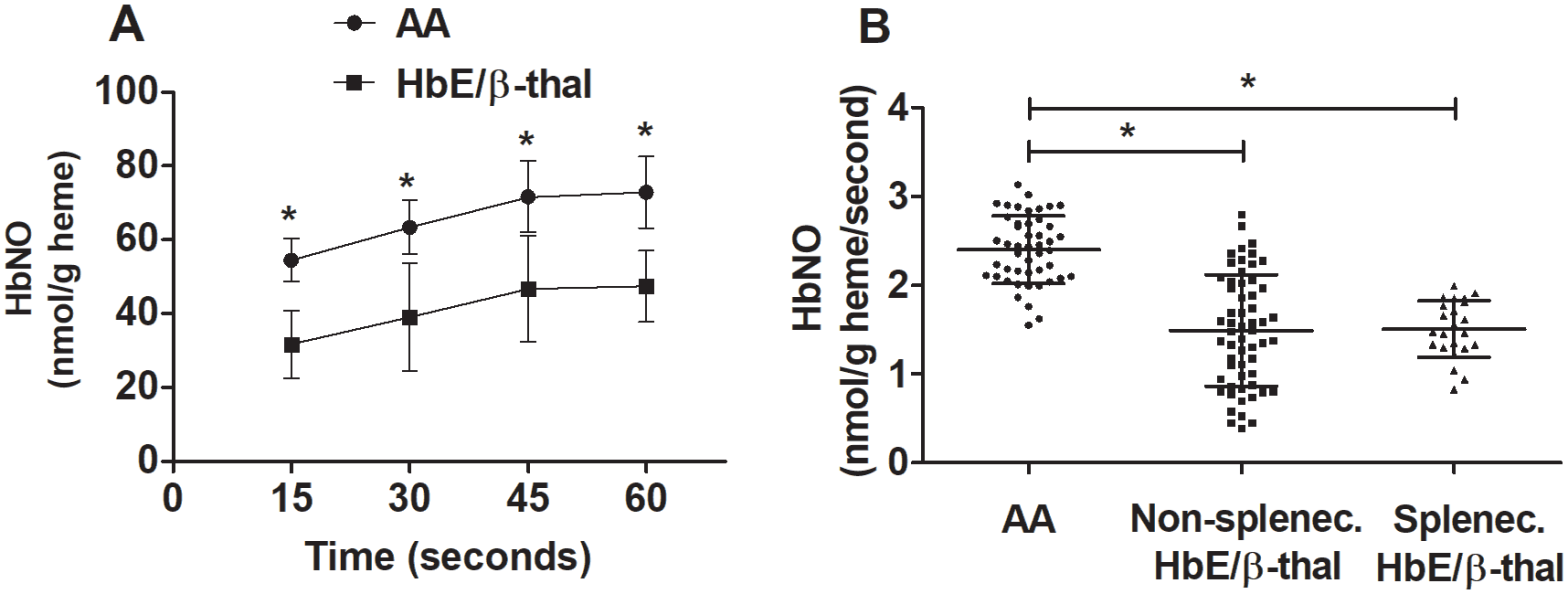
Decreased HbNO production in HbE/ß-thal subjects. (A) HbNO production from the reaction between 0.5 μM nitrite and deoxyHb dialysates (50 μM heme) of normal subjects (AA, n = 10) and non-splenectomized HbE/ß-thal subjects (n = 10) as a function of time. HbNO was measured by the chemiluminescence method. *P < 0.001 by unpaired *t*-test. Data are means ± SD. (B) Rate of HbNO production was measured at 30 seconds from the reactions between 0.5 μM nitrite and deoxyHb (50 μM heme) dialysates from normal subjects (n = 47), non-splenectomized HbE/ß-thal subjects (n = 58), and splenectomized HbE/ß-thal subjects (n = 23). *P < 0.0001, one-way ANOVA with Bonferroni post hoc test. Lines represent means ± SD.

By spectral deconvolution, the amount of HbNO generated after 30-second incubation of nitrite with deoxyHb obtained from healthy and non-splenectomized HbE/ß-thal was 42 and 25%, respectively. By chemiluminescence, HbNO production at times 15, 30, 45 and 60 seconds was lower in HbE/ß-thal subjects than healthy subjects (Fig 1A). DeoxyHb dialysates of non-splectomized and splenectomized HbE/ß-thal subjects exhibited lower nitrite reductase activity than those of healthy subjects (mean ± SD: 1.49 ± 0.63, 1.50 ± 0.32, and 2.40 ± 0.38 nmol/g heme/second, respectively; *P* < 0.0001) (Fig 1B). To test whether hemoglobin dialysate of HbE/ß-thal subjects had reduced ability to bind NO, diethylamine NONOate (DEA NONOate) was used. Incubation of hemoglobin dialysate (50 μM) from normal and non-splenectomized HbE/ß-thal subjects with DEA NONOate (0.5 μM, half-life of 2 min at 37°C) for 8 min resulted in equivalent amount of HbNO formation (63.5 ± 10.9 and 59.3 ± 9.6 nmol/g heme, respectively, n = 10 each, *P* = 0.43 by unpaired *t*-test).

### Platelet activation

**Fig 2:**
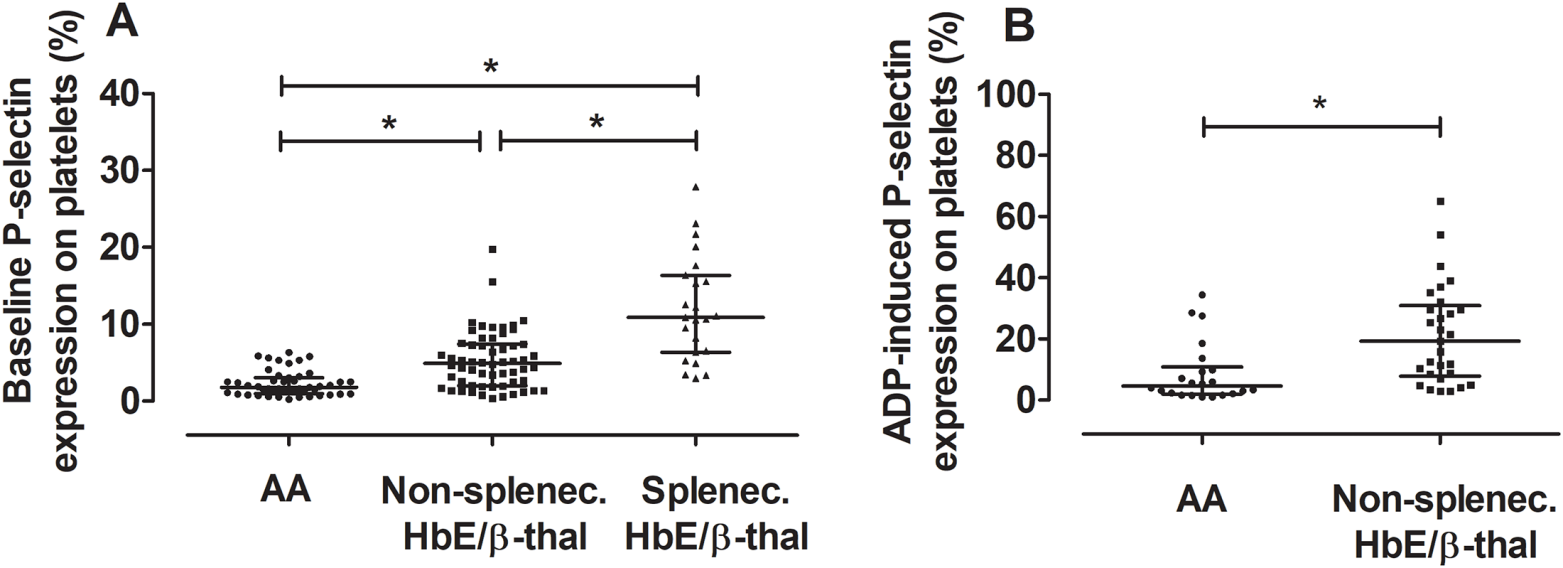
P-selectin expression at baseline and in response to stimulation with ADP. (A) Non-splenectomized and splenectomized HbE/ß-thal subjects (n = 58 and 23, respectively) had higher baseline platelet P-selectin expression than normal subjects (AA, n = 47). Splenectomized HbE/ß-thal subjects also had higher baseline platelet P-selectin expression than non-splenectomized HbE/ß-thal subjects. *P < 0.0001 by Kruskal-Wallis test. Lines represent medians and interquartile range. (B) Non-splenectomized HbE/ß-thal subjects (n = 29) had higher platelet reactivity to stimulation with ADP (1 μM) than normal subjects (n = 22). *P = 0.0007 by Mann-Whitney U test. Lines represent medians and interquartile range.

Increased percentages of baseline P-selectin (CD62P)-expressed platelets were observed in non-splenectomized and splenectomized HbE/ß-thal subjects when compared with normal subjects: median (interquartile range) 4.90 (1.99–7.43)%, 10.87 (6.36–16.36)%, and 1.80 (0.95–3.00)%, respectively; *P* < 0.0001 (Fig 2A). Similarly, the percentages of P-selectin-expressed platelets in response to ADP stimulation were elevated in HbE/ß-thal subjects when compared with normal subjects: median (interquartile range) 19.3 (7.80–30.90)% versus 4.60 (1.97–10.85)%, respectively; *P* = 0.0007 (Fig 2B).

### Correlation analysis

**Fig 3:**
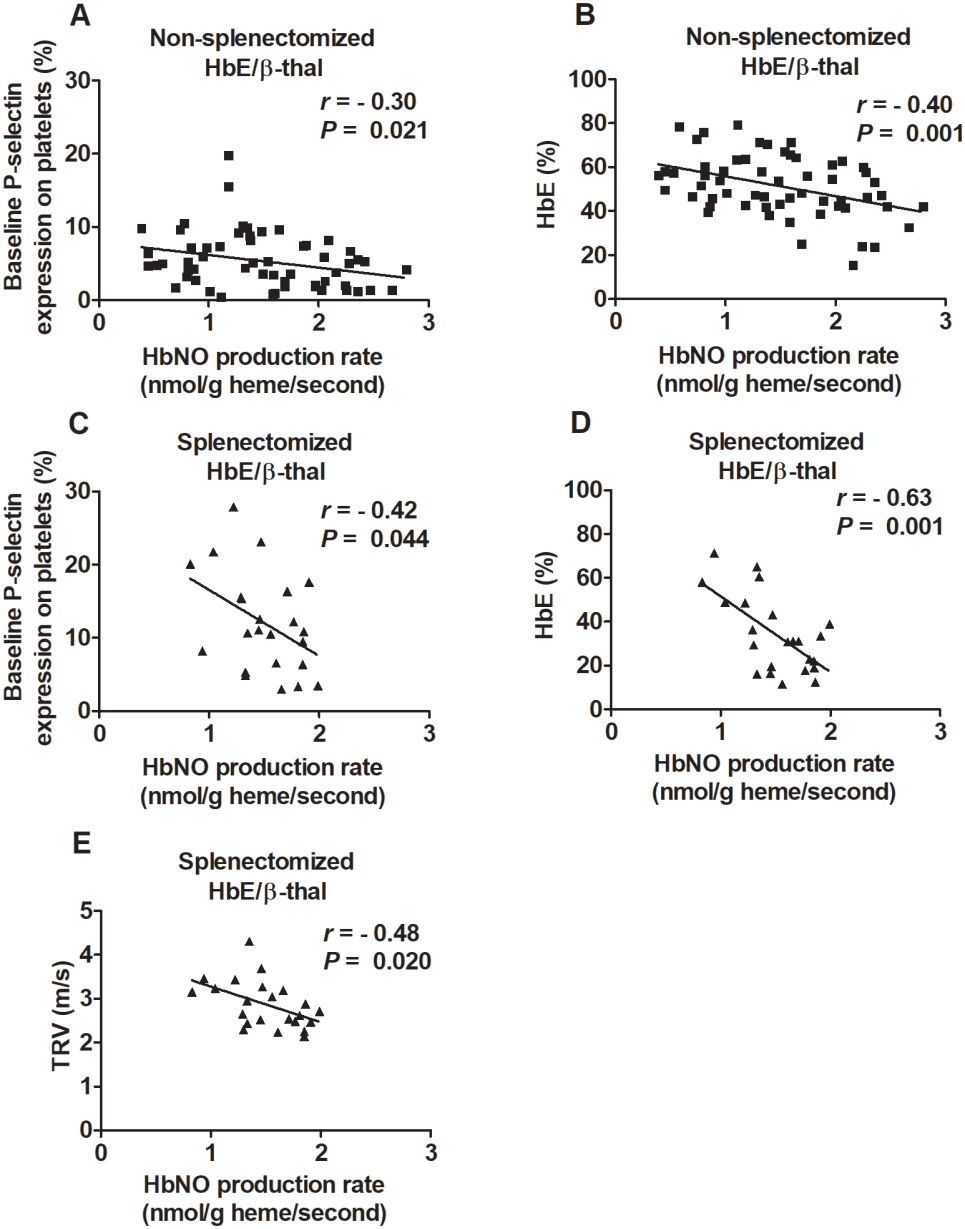
Correlations. Negative correlations between HbNO production rate and baseline platelet P-selectin expression (A) and HbE levels (B) in non-splenectomized HbE/ß-thal subjects (n = 58, by Spearman and Pearson correlation methods, respectively). Negative correlations between HbNO production rate and baseline platelet P-selectin expression (C) and HbE levels (D) in splenectomized HbE/ß-thal subjects (n = 30, by Pearson correlation methods). (E) Negative correlations between HbNO production rate and tricuspid regurgitant velocity (TRV) in splenectomized HbE/ß-thal subjects (n = 23, by Pearson correlation method).

In both non-splenectomized and splenectomized HbE/ß-thal subjects, HbNO production rate showed negative correlation with baseline P-selectin expression on platelets and HbE levels (Fig 3A–3D). There was no correlation between HbNO production rate and the percentages of ADP (1 μM)-induced platelet P-selectin expression. Hemoglobin A levels exhibited a positive correlation with HbNO production rate (*r* = 0.37, *P* = 0.005). In splenectomized HbE/ß-thal subjects, TRV exhibited negative correlation with HbNO production rate (Fig 3E). There was no correlation between hemoglobin F and nitrite reductase activity. TRV and HbNO production rate showed no correlation with hemolytic markers (hemoglobin and indirect bilirubin).

### Platelet inhibition by nitrite plus deoxygenated erythrocytes

**Fig 4:**
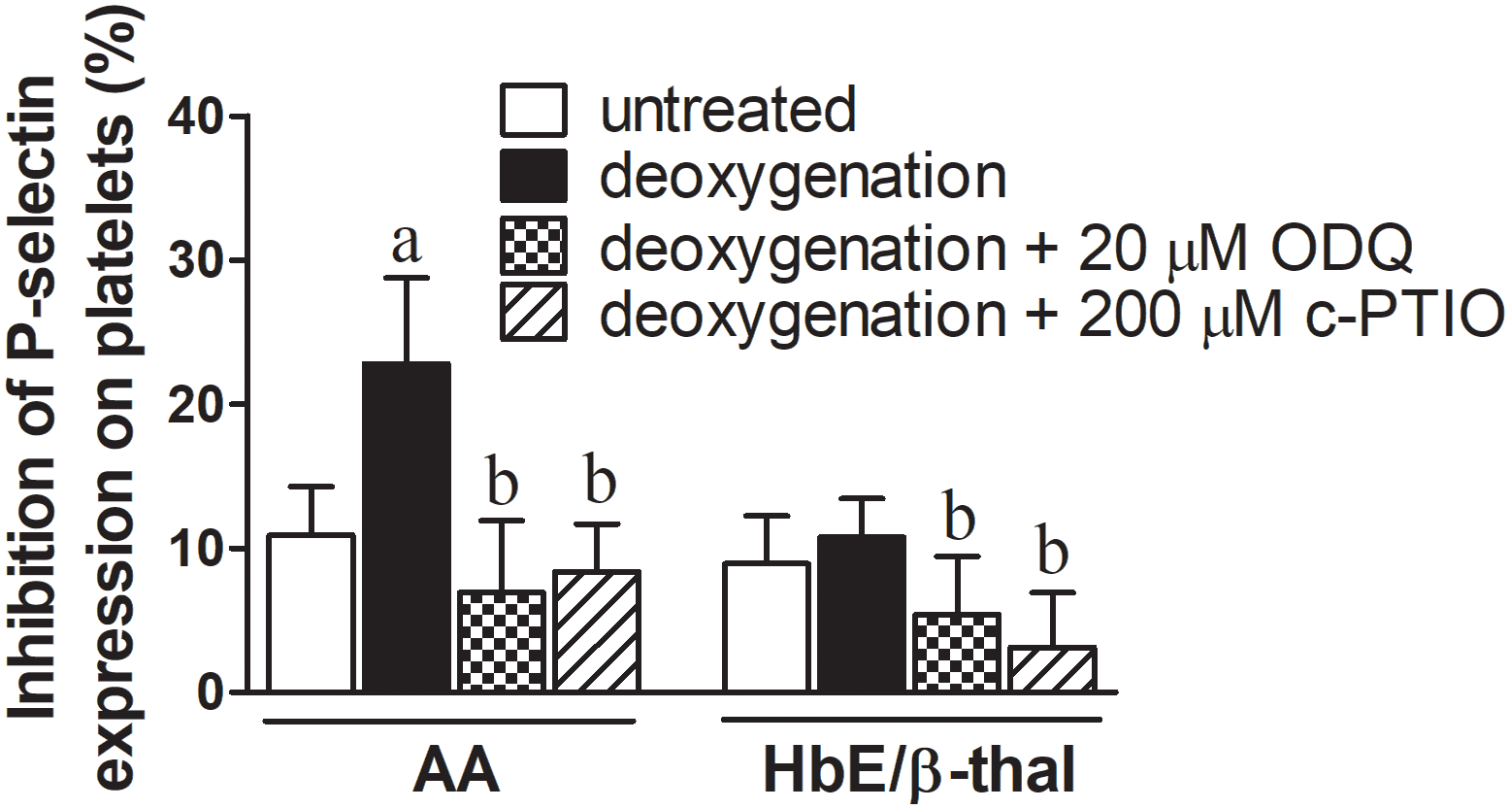
Inhibition of ADP-induced P-selectin expression on platelets by deoxygenated erythrocytes + nitrite *in vitro*. Deoxygenated erythrocytes (20% hematocrit in PBS) from normal (AA) and HbE/ß-thal subjects were incubated with 0.5 μM sodium nitrite at 37 °C in PBS pH 7.4 for 10 min. Afterwards, activation of platelets obtained from normal subjects was induced by 10-min incubation with 20 μM ADP. 20 μM ODQ or 200 μM C-PTIO were used as soluble guanylate cyclase inhibitor and NO scavenger, respectively. ^a^*P* < 0.01 compared with untreated erythrocytes, ^b^*P* < 0.01 compared with deoxygenated erythrocytes. (ANOVA with Bonferroni post hoc test). Data are means ± SD. (n = 5–11 and 5–9 for healthy and HbE/ß-thal, respectively).

In the presence of 0.5 μM nitrite, untreated erythrocytes (no deoxygenation) of normal subjects could inhibit ADP-induced platelet activation by 10.92 ± 3.38% (Fig 4). Deoxygenation of erythrocytes increased the %inhibition to 22.79 ± 5.95. The inhibition of P-selectin expression by deoxygenated erythrocytes + nitrite was abolished by 20 μM ODQ or 200 μM C-PTIO (mean ± SD: 6.94 ± 4.98% and 8.36 ± 3.32%, respectively). Untreated erythrocytes from HbE/ß-thal subjects could inhibit ADP-induced platelet activation by 8.94 ± 3.32%. Deoxygenation of HbE/ß-thal erythrocytes had no effect on ADP-induced P-selectin expression (%inhibition = 10.79 ± 2.68).

## Discussion

In this study, we present a functional consequence of the presence of HbE in erythrocytes, *i*.*e*. a decrease in nitrite reductase activity of deoxyHb from erythrocytes of HbE/ß-thal subjects as demonstrated by decreased HbNO. The decreased HbNO formation was less likely due to reduction of HbE binding to NO because of the equivalent HbNO formation from reactions of DEA NONOate with normal and HbE/ß-thal hemoglobin dialysate. Significantly, to the best of our knowledge, this is the first finding to demonstrate that HbE dysfunction as a nitrite reductase [13] occurs in erythrocytes and correlates with baseline enhanced platelet activation in HbE/ß-thal subjects compared to healthy subjects. Baseline P-selectin expression on platelets and HbE levels showed a negative correlation with HbNO formation rate, suggesting an association of NO generated from the deoxyHb nitrite reductase activity with platelet activity. Our *in vitro* experiments showed that nitrite plus deoxygenated erythrocytes from HbE/ß-thal subjects could not inhibit ADP-induced platelet activation under these conditions where normal hemoglobin in red cells do so.

P-selectin expression serves as a degranulation marker occurring during platelet activation. P-selectin has a supporting role in platelet rolling on activated endothelial cells [26]. Adherent platelets undergo activation during which the granule contents are released. During the release reaction, large amount of mediators, including ADP, calcium, ATP, serotonin, von Willebrand factor, and platelet factor 4 are actively secreted from granules. ADP secreted from granules is important in mediating the next steps of activation and aggregation, causing platelets to adhere to one another. Thalassemia patients have increased P-selectin expression on platelets and soluble P-selectin in plasma [17,24,27]. Herein, we provide additional data on the association of nitrite reductase activity of HbE with P-selectin expression on platelets.

HbE/ß-thal subjects manifest endothelial dysfunction relevant to oxidative stress and reduced production of NO through the endothelial nitric oxide synthase (eNOS) pathway [10]. Decreased NO availability and activity such as decreased levels, uncoupling eNOS, or signaling can disturb vascular homeostasis, including abnormal local vasoconstriction, increased platelet activation/aggregation, macrophage infiltration/activation, endothelial cell activation, and vascular remodeling [8]. Alteration in NO is associated with vascular complications in thalassemia such as pulmonary hypertension [28]; however, it is unclear whether it contributes to the complications or only reflects consequence of vascular abnormality. Our findings of a decreased nitrite reductase activity of HbE in HbE/ß-thal erythrocytes and its correlation with increased platelet activity suggest that HbE plays a role in the pathophysiology of HbE/ß-thalassemia.

As stated earlier, it has been established *in vitro* that deoxyHb can catalyze the reduction of nitrite to NO via the nitrite reductase activity under hypoxic conditions, which contributes to hypoxic vasodilation [7]. The reductive reaction between deoxyHb and nitrite yields methemoglobin and NO that can further bind with deoxyHb to form HbNO [29]. We show here that deoxyHb from HbE/ß-thal subjects has decreased nitrite reductase activity relative to hemoglobin (HbA) from healthy subjects. The decrease in nitrite reductase activity of HbE/ß-thal hemoglobin is consistent with a report that purified hemoglobin E has 2.5 fold decrease in nitrite reductase activity compared to hemoglobin A due to its altered redox status [13].

Nitrite inhibits platelet activity in the presence of erythrocytes, in particular under deoxygenation [15,30]. Nitrite plus deoxygenated erythrocytes increases phosphorylated vasodilator-stimulated phosphoprotein in platelets, demonstrating that the effect of nitrite is mediated, at least in part, through the NO/cGMP/protein kinase G pathway [31,32]. DeoxyHb in erythrocytes is proposed to reduce nitrite to NO, and it has been proposed that NO could be exported out of erythrocytes to exert biological activities [33]. In our study, the diminished platelet inhibition by nitrite in the presence of erythrocytes from HbE/ß-thal subjects confirmed the findings of decreased NO generation from nitrite, and implies the possibility that impaired HbE-catalyzed production of NO might contribute to platelet hyperactivation. In ß-thalassemia, the increased platelet activation is associated with the increase in estimated pulmonary artery pressure measured by echocardiography [15,17].

Unexpectedly, we found that HbE/ß-thal adult subjects had higher blood nitrite than healthy subjects despite endothelial dysfunction [34]. Nitrite is continuously produced by vascular endothelium. Nitrite is shown to be a marker of endothelial function and correlated with cardiovascular risk factors [35]. Blood nitrite was reported to be lower in HbE/ß-thal children, which was associated with clinical severity, higher levels of cell-free hemoglobin, and an enhanced degree of hemolysis [9]. Acetylcholine-induced vasodilation was impaired in ß-thalassemic mice despite eNOS overexpression, suggesting endothelial dysfunction and eNOS uncoupling [34]. However, the elevated blood nitrite found in our study could be explained by multiple factors, including ingestion of nitrite/nitrate-rich diet [36], inability of HbE to efficiently convert nitrite to NO compared to HbA [13], increased inducible NO synthase (iNOS) expression [37], and medications that enhance eNOS activity. Our study was limited by lack of diet control. It should be point out here that the iNOS isoform is normally absent in vascular endothelial cells under physiologic condition. Expression of iNOS is induced in blood vessels under pathologic conditions such as inflammation and oxidative stress [38].

Certain drugs such as hydroxyurea, deferoxamine, and erythropoietin can increase eNOS activity [39–41]. Hydroxyurea is used in patients with thalassemia and sickle cell disease to increase fetal hemoglobin. Hydroxyurea was demonstrated to increase NO production in sickle cell patients [42], animals [43], and *in vitro* culture of endothelial cells [39]. NO is generated during biotransformation of hydroxyurea in liver, and believed to be a molecule mediating fetal hemoglobin induction [44]. Moreover, hydroxyurea was shown to stimulate eNOS in endothelial cells. Hydroxyurea, deferoxamine and erythropoietin increase NO production in endothelial cells by eNOS stimulation via phosphorylation of Ser1177 which is related to the activation of phosphoinositide 3-kinase/protein kinase pathway. In a mouse model of cardiac ischemia-reperfusion injury, erythropoietin stimulated eNOS phosphorylation and NO production in coronary artery endothelial cells lead to the attenuation of myocardial infarct size [41]. Increased serum erythropoietin was reported in thalassemia patients [45]; which thereby might account for the increased nitrite blood levels.

In conclusion, deoxyHb dialysates from HbE/ß-thal erythrocytes exhibit a decreased ability to produce NO from nitrite as a result of a decreased nitrite reductase activity of HbE. Since decreased nitrite reductase activity of deoxyHb may lead to NO deficiency locally at the site of tissue hypoxia regardless of elevated circulating nitrite levels as induced by multiple factors. This intrinsic loss of HbE function has been recently shown in culture to aggravate endothelial dysfunction [46] and; as indicated in this paper, may contribute in part to an increase in platelet activation and vascular complications in HbE/ß-thalassemia. Further research is needed to verify other possible mechanisms of increased platelet activity in thalassemia, including decreased NO synthesis in platelets or alteration in intracellular signaling pathways.

## Supporting information

S1 Table Demographic and laboratory data. Baseline data of HbE/ß-thal subjects.
(PDF)

S2 Table HbNO production in HbE/ß-thal subjects. Data of HbNO production from the reaction between nitrite and deoxyHb dialysates of normal healthy subjects and non-splenectomized HbE/ß-thal subjects as a function of time.
(PDF)

S3 Table HbNO production and P-selectin expression on platelets. Data of HbNO production rate from the reactions between nitrite and deoxyHb dialysates, P-selectin expression on platelets at baseline and in response to stimulation with ADP, HbE, and HbF levels.
(PDF)

S4 Table Inhibition of ADP-induced P-selectin expression on platelets by deoxygenated erythrocytes + nitrite *in vitro*. Data of *in vitro* experiments showing the effects of deoxygenated erythrocytes from normal and HbE/ß-thal on P-selectin expression on platelets.
(PDF)
