# Supplementary material for: Decreased nitrite reductase activity of deoxyhemoglobin correlates with platelet activation in hemoglobin E/ß-thalassemia subjects
Source: PLoS One. 2018 Sep 20;13(9):e0203955. doi: 10.1371/journal.pone.0203955 (PMC6147434; doi:10.1371/journal.pone.0203955)
Supplement: S1 Table — Baseline data of HbE/ß-thal subjects. (PDF) [file pone.0203955.s001.pdf]

Normal Subjects	Age (years)	Sex (Male or Female)	Hb (g/dL)	Hematocrit (%)	White blood cell count ($\times10^9$/L)	Platelet count ($\times10^3$/mL)
H01	23	Male	14.5	37.6	6.8	275
H02	41	Female	12.4	32.2	6.2	322
H03	39	Female	10.3	38.2	6.1	427
H04	23	Male	14.4	42.1	6.4	116
H05	27	Male	13.3	45.8	7.1	251
H06	24	Male	14.5	50.4	7.1	160
H07	23	Male	15.6	41.2	7.6	165
H08	25	Male	14.3	39.4	7.3	273
H09	27	Male	13.5	35.8	6.4	331
H10	27	Female	10.8	38.3	8.0	208
H11	25	Female	13.6	42.1	5.4	236
H12	24	Male	14.5	36.6	7.5	285
H13	25	Female	12.8	34.4	6.7	207
H14	24	Male	10.1	41.4	4.8	232
H15	25	Male	14.1	42.9	5.0	205
H16	34	Female	12.5	40.1	5.5	305
H17	56	Female	10.4	37.1	6.1	375
H18	48	Female	13.7	31.4	8.3	245
H19	54	Female	14.0	42.2	5.9	270
H20	40	Female	15.9	42.5	8.8	241
H21	57	Male	12.5	48.5	6.0	203
H22	42	Male	13.3	39.2	6.4	216
H23	31	Female	11.1	39.2	7.5	239
H24	55	Female	12.3	35.9	6.1	222
H25	37	Female	13.6	38.1	9.0	179
H26	23	Female	14.5	41.6	4.6	247
H27	34	Male	12.7	43.7	7.1	243
H28	39	Female	11.5	38.6	8.7	336
H29	31	Female	13.4	36.8	7.6	296
H30	27	Female	11.3	34.9	5.8	276
H31	26	Female	12.6	38.7	4.4	276
H32	33	Female	14.5	43.1	5.7	183
H33	28	Male	16.3	48.2	6.4	273
H34	25	Male	13.9	41.6	8.3	198
H35	28	Female	13.7	42.5	5.8	220
H36	37	Female	12.0	36.7	5.9	170
H37	24	Female	12.8	40.3	8.1	323
H38	26	Female	11.0	33.9	4.8	313
H39	24	Female	15.3	45.2	7.0	211
H40	27	Female	11.9	36.0	12.1	340
H41	28	Female	14.1	44.3	8.6	276
H42	26	Male	14.2	43.0	7.0	274

H43	32	Male	12.1	36.9	7.3	260
H44	25	Male	16.0	47.4	8.9	282
H45	31	Male	15.4	46.8	9.0	193
H46	25	Male	12.3	37.3	5.4	324
H47	26	Male	14.2	43.2	7.2	265

Non-splenectomized HbE/β-thal Subjects	Age (years)	Sex (Male or Female)	Hb (g/dL)	Hematocrit (%)	White blood cell count ($\times 10^9$/L)	Platelet count ($\times 10^3$/mL)
NSP01	34	Male	7.1	21.0	6.8	156
NSP02	41	Male	8.1	26.8	7.1	179
NSP03	36	Male	9.7	28.6	6.2	185
NSP04	35	Female	8.6	27.3	6.7	162
NSP05	36	Female	7.7	23.5	5.3	132
NSP06	31	Female	6.3	19.5	7.7	122
NSP07	44	Female	7.1	24.8	3.3	116
NSP08	36	Male	8.5	27.0	3.6	186
NSP09	24	Male	7.0	27.0	7.7	141
NSP10	46	Male	6.9	24.2	7.2	102
NSP11	22	Male	7.3	27.5	8.0	174
NSP12	44	Male	7.0	24.9	5.5	269
NSP13	28	Female	11.2	36.3	4.6	84
NSP14	33	Female	9.2	28.0	7.2	278
NSP15	41	Male	7.9	25.0	6.4	153
NSP16	22	Female	8.3	29.0	11.8	102
NSP17	39	Female	6.4	20.0	4.7	99
NSP18	43	Male	7.6	24.7	4.5	201
NSP19	38	Male	6.2	20.0	5.3	82
NSP20	33	Female	7.0	22.0	5.6	111
NSP21	33	Male	6.4	20.0	4.2	227
NSP22	51	Female	4.9	16.0	4.7	143
NSP23	28	Female	7.8	25.0	6.8	374
NSP24	47	Female	9.0	28.0	11.0	386
NSP25	29	Female	8.2	5.0	6.3	122
NSP26	35	Male	7.9	6.0	6.8	122
NSP27	22	Male	7.1	22.0	8.5	166
NSP28	41	Male	8.6	27.0	6.2	208
NSP29	31	Female	7.7	24.0	7.5	324
NSP30	47	Male	6.6	21.0	5.1	312
NSP31	41	Female	6.4	21.0	6.7	115
NSP32	38	Female	6.2	21.0	3.8	103
NSP33	32	Male	7.5	23.0	6.8	334
NSP34	19	Female	7.0	22.4	7.8	240
NSP35	19	Male	6.3	22.0	6.0	182

NSP36	31	Female	9.5	30.0	5.6	114
NSP37	23	Female	7.7	24.0	7.3	210
NSP38	21	Male	7.7	25.0	7.6	247
NSP39	26	Male	5.7	20.0	7.4	178
NSP40	25	Female	6.4	21.0	7.2	238
NSP41	32	Male	6.7	21.0	5.9	181
NSP42	33	Female	5.4	18.0	5.4	129
NSP43	37	Male	5.3	16.0	1.0	565
NSP44	36	Female	7.2	24.0	14.2	224
NSP45	21	Male	9.5	30.8	6.7	308
NSP46	43	Male	6.1	22.1	7.5	221
NSP47	39	Male	8.5	26.5	8.2	160
NSP48	45	Female	9.5	29.0	9.3	276
NSP49	43	Female	10.3	34.8	8.1	231
NSP50	25	Female	7.2	24.7	5.6	181
NSP51	20	Female	10.0	31.4	6.8	140
NSP52	36	Female	7.0	24.3	5.9	153
NSP53	18	Female	10.1	34.5	6.4	321
NSP54	21	Female	8.4	30.1	8.6	284
NSP55	23	Male	7.8	24.7	5.4	239
NSP56	21	Female	8.8	30.0	10.3	303
NSP57	39	Male	6.9	24.1	15.2	207
NSP58	36	Female	5.6	17.8	5.5	92

Splenectomized HbE/β-thal Subjects	Age (years)	Sex (Male or Female)	Hb (g/dL)	Hematocrit (%)	White blood cell count ($\times 10^9$/L)	Platelet count ($\times 10^3$/mL)
SP01	35	Female	7.7	22.7	10.6	635
SP02	25	Female	4.9	14.5	10.9	670
SP03	21	Female	8.0	23.6	11.7	718
SP04	23	Male	7.8	23.5	22.5	1026
SP05	22	Female	9.8	23.0	8.8	398
SP06	17	Female	7.6	30.9	11.1	287
SP07	22	Male	10.5	20.3	6.5	541
SP08	47	Female	7.5	22.0	10.7	517
SP09	26	Male	7.5	22.0	20.5	437
SP10	22	Male	9.0	23.5	6.0	552
SP11	49	Female	6.3	18.4	17.3	673
SP12	37	Female	7.1	21.0	7.5	551
SP13	44	Male	7.5	21.0	8.8	580
SP14	41	Female	7.7	22.1	5.7	853
SP15	34	Male	6.9	22.6	6.5	739
SP16	20	Female	8.6	21.4	7.5	967
SP17	24	Female	9.1	20.2	12.2	547
SP18	45	Male	6.2	25.3	5.0	642

SP19	47	Male	5.4	29.2	7.6	708
SP20	25	Female	5.0	21.0	12.4	622
SP21	23	Female	6.9	17.0	13.7	261
SP22	25	Male	6.7	26.1	19.1	605
SP23	22	Male	7.8	20.3	11.6	551

<table>
<tr><th>Aspartate aminotransferase (U/L)</th><th>Blood urea nitrogen (mg/dL)</th><th>Creatinine (mg/dL)</th><th>Indirect bilirubin (mg/dL)</th><th>Blood nitrite (nM)</th></tr>
<tr><td rowspan="12">N/A</td><td rowspan="12">N/A</td><td rowspan="12">N/A</td><td rowspan="9">N/A</td><td>124.19</td></tr>
<tr><td>163.6</td></tr>
<tr><td>76.54</td></tr>
<tr><td>131.63</td></tr>
<tr><td>109.9</td></tr>
<tr><td>83.93</td></tr>
<tr><td>120.46</td></tr>
<tr><td>96.36</td></tr>
<tr><td>104.61</td></tr>
<tr><td>0.4</td><td>218.25</td></tr>
<tr><td>0.5</td><td>207.09</td></tr>
<tr><td>0.8</td><td>207.23</td></tr>
<tr><td>20</td><td>10</td><td>0.8</td><td>0.2</td><td>183.41</td></tr>
<tr><td>28</td><td>7</td><td>0.41</td><td>0.1</td><td>144.89</td></tr>
<tr><td>23</td><td>9</td><td>0.66</td><td>0.8</td><td>94.83</td></tr>
<tr><td>18</td><td>7</td><td>0.54</td><td>0.2</td><td>76.37</td></tr>
<tr><td>17</td><td>8</td><td>0.54</td><td>0.3</td><td>100.21</td></tr>
<tr><td>15</td><td>13</td><td>0.59</td><td>0.2</td><td>147.8</td></tr>
<tr><td>23</td><td>13</td><td>1.05</td><td>0.6</td><td>204.47</td></tr>
<tr><td>20</td><td>9</td><td>0.8</td><td>0.4</td><td>250.34</td></tr>
<tr><td>57</td><td>9</td><td>0.6</td><td>0.8</td><td>222.78</td></tr>
<tr><td>27</td><td>11</td><td>0.69</td><td>0.4</td><td>180.51</td></tr>
<tr><td>27</td><td>11</td><td>0.63</td><td>0.5</td><td>197.45</td></tr>
<tr><td>15</td><td>8</td><td>0.73</td><td>0.6</td><td>206.6</td></tr>
<tr><td>17</td><td>6</td><td>0.59</td><td>0.4</td><td>264.27</td></tr>
<tr><td>20</td><td>6</td><td>0.51</td><td>0.2</td><td>188.86</td></tr>
<tr><td>17</td><td>20</td><td>0.77</td><td>0.4</td><td>199.67</td></tr>
<tr><td>20</td><td>18</td><td>1.2</td><td>0.2</td><td>186.34</td></tr>
<tr><td>22</td><td>16</td><td>0.91</td><td>0.1</td><td>176.87</td></tr>
<tr><td>13</td><td>10</td><td>0.59</td><td>0.4</td><td>168.43</td></tr>
<tr><td>19</td><td>10</td><td>1.08</td><td>0.1</td><td>105.35</td></tr>
<tr><td>22</td><td>11</td><td>1.1</td><td>0.3</td><td>175.89</td></tr>
<tr><td>16</td><td>8</td><td>0.91</td><td>0.2</td><td>184.25</td></tr>
<tr><td>29</td><td>12</td><td>0.95</td><td>0.2</td><td>204.27</td></tr>
<tr><td>16</td><td>8</td><td>0.61</td><td>0.4</td><td>218.14</td></tr>
<tr><td>15</td><td>12</td><td>0.72</td><td>0.3</td><td rowspan="7">N/A</td></tr>
<tr><td>16</td><td>10</td><td>0.89</td><td>0.4</td></tr>
<tr><td>27</td><td>15</td><td>1.14</td><td>0.2</td></tr>
<tr><td>16</td><td>6</td><td>0.77</td><td>0.6</td></tr>
<tr><td>17</td><td>7</td><td>0.8</td><td>0.3</td></tr>
<tr><td>24</td><td>14</td><td>0.58</td><td>0.3</td></tr>
<tr><td>23</td><td>17</td><td>0.75</td><td>0.4</td></tr>
</table>

27	8	1.06	0.1	
27	11	0.94	0.5	
28	6	0.88	0.4	
26	16	0.63	0.2	
29	7	0.6	0.5	

Aspartate aminotransferase (U/L)	Blood urea nitrogen (mg/dL)	Creatinine (mg/dL)	Ferritin (ng/mL)	Indirect bilirubin (mg/dL)	Duration from last transfusion (days)	Blood nitrite (nM)
N/A	N/A	N/A	213.1	4.5	30	181.02
			2298.6	4.5	180	70.00
			1709.5	0.9	365	62.93
			2842.5	0.9	72	329.33
			2188.2	1.2	N/A	220.44
			5303.3	1.6	120	324.74
			1980.2	0.1	N/A	215.00
			375.6	6.5		295.00
			632.2	1.2	20	274.81
			606.6	9.1	N/A	249.17
			4311.8	1.2		283.17
			814.0	3.8	14	85.00
			983.3	2.7	365	101.00
			193.0	1.3	300	310.27
			331.4	1.8	N/A	280.15
			584.1	1.4		189.30
			406.0	2.3		207.00
			336.0	2.0		89.38
			1775.0	1.5		190.34
			1500.0	3.7		168.30
			446.0	5.9		285.40
			356.0	6.8		320.78
			768.0	3.9		333.67
45	10	1.04	207.2	0.8		279.74
37	14	0.48	280.0	2.6		346.08
36	9	0.38	1798.0	1.5		343.55
69	9	0.45	97.6	4.6		
50	11	0.39	634.0	2.1		
27	14	0.71	1836.0	2.6		
22	9	0.69	399.0	0.6		
29	10	0.62	429.7	3.4		
73	14	0.40	1154.2	1.4		
18	8	0.41	286.5	1.3		
37	15	0.63	337.3	3.7		
56	12	0.31	1474.0	1.7		

47	12	0.57	643.1	2.2	1460	N/A
37	12	0.39	1500.0	2.3	N/A	
30	10	0.49	1484.0	4.5		
36	10	0.42	345.0	3.3	45	
37	15	0.78	615.6	4.2	N/A	
32	29	0.86	902.3	2.1	365	
20	9	0.44	854.2	6.6	N/A	
55	7	0.44	1409.9	2.7		
39	7	0.43	720.0	1.3		
24	11	0.67	68.4	1.3		
34	12	0.45	2746.0	2.5		
66	10	0.58	6885.3	3.3		
20	9	0.52	567.9	2.0	90	
18	9	0.72	335.1	0.5	N/A	
55	12	0.51	614.9	4.9		
32	19	0.69	198.1	4.6		
53	13	0.73	1049.4	3.9	90	
28	16	0.70	155.5	2.3	N/A	
23	11	0.66	580.6	1.7		
28	13	0.60	264.4	4.7	720	
17	12	0.59	272.3	1.3	N/A	
97	15	0.74	3462.4	3.9		
92	18	0.58	8061.3	2.0	300	

Aspartate aminotransferase (U/L)	Blood urea nitrogen (mg/dL)	Creatinine (mg/dL)	Ferritin (ng/mL)	Indirect bilirubin (mg/dL)	Duration of splenectoy (years)	Duration from last transfusion (days)	Blood nitrite (nM)
N/A			1200.0	3.3	26	N/A	107.56
			180.0	2.1	23	16	173.52
			2575.4	1.6	16	21	164.87
			1428.2	3.5	12	16	185.41
			217.5	3.4	19	23	206.08
			3191.9	4.5	4	21	271.96
			2500.2	4.4	19	30	249.83
			213.1	2.6	32	25	116.96
24	14	0.50	378.2	3.7	7	N/A	383.68
69	11	0.40	2775.0	3.0	17		403.86
80	12	0.30	1919.4	1.6	49	37	294.74
56	17	0.70	2400.0	3.9	36	60	143.21
106	9	0.20	3627.0	3.6	24	36	241.43
47	11	0.20	2798.6	2.4	31	30	149.51
19	10	0.40	432.2	3.9	18	14	297.50
42	10	0.50	606.6	4.2	13	71	163.87
25	7	0.40	4311.8	2.0	5	60	122.87
30	29	1.60	814.0	6.3	28	720	424.25

87	11	0.32	983.3	2.5	39	90	304.84
56	10	0.61	1200.0	4.7	23	120	187.82
39	8	0.59	1567.0	3.5	18	N/A	181.77
109	12	0.42	747.5	3.9	17	60	120.61
83	10	0.42	1680.3	2.5	17	21	113.96
