# Supplementary material for: Decreased nitrite reductase activity of deoxyhemoglobin correlates with platelet activation in hemoglobin E/ß-thalassemia subjects
Source: PLoS One. 2018 Sep 20;13(9):e0203955. doi: 10.1371/journal.pone.0203955 (PMC6147434; doi:10.1371/journal.pone.0203955)
Supplement: S2 Table — Data of HbNO production from the reaction between nitrite and deoxyHb dialysates of normal healthy subjects and non-splenectomized HbE/ß-thal subjects as a function of time. (PDF) [file pone.0203955.s002.pdf]

Healthy Subject	HbNO production (nmol/g heme)			
	reaction time 15 sec	reaction time 30 sec	reaction time 45 sec	reaction time 60 sec
H01	53.15	68.53	93.95	94.11
H05	49.45	71.49	63.88	63.57
H07	48.06	56.82	77.46	78.17
H08	62.30	58.82	77.46	78.17
H09	62.02	73.49	63.88	63.57
H10	50.28	64.79	72.06	74.01
H12	49.52	52.81	63.40	64.00
H24	59.00	60.79	72.06	74.01
H26	60.67	55.81	63.40	63.99
H27	50.53	70.34	68.00	74.00

HbE/β-thal Subject	HbNO production (nmol/g heme)			
	reaction time 15 sec	reaction time 30 sec	reaction time 45 sec	reaction time 60 sec
NSP01	43.57	56.70	50.63	72.87
NSP02	40.78	42.00	46.26	42.79
NSP03	37.33	47.91	69.59	48.67
NSP12	35.33	50.79	67.58	44.67
NSP15	32.64	47.49	44.41	39.20
NSP16	15.50	13.50	20.74	39.57
NSP17	17.50	13.50	34.57	48.34
NSP18	32.64	40.47	47.03	49.41
NSP25	31.21	39.90	42.00	44.22
NSP26	30.21	38.10	43.78	44.22
