# Supplementary material for: Decreased nitrite reductase activity of deoxyhemoglobin correlates with platelet activation in hemoglobin E/ß-thalassemia subjects
Source: PLoS One. 2018 Sep 20;13(9):e0203955. doi: 10.1371/journal.pone.0203955 (PMC6147434; doi:10.1371/journal.pone.0203955)
Supplement: S3 Table — Data of HbNO production rate from the reactions between nitrite and deoxyHb dialysates, P-selectin expression on platelets at baseline and in response to stimulation with ADP, HbE, and HbF levels. (PDF) [file pone.0203955.s003.pdf]

| Healthy Subject | HbNO production at 30 sec (nmol/g heme/sec) | Baseline Plt P-selectin (%) | 1 μM ADP-induced Plt P-selectin (%) | HbE (%) |
|---|---|---|---|---|
| H01 | 2.28 | 5.87 | N/A | 0.0 |
| H02 | 2.43 | 2.46 | | 0.0 |
| H03 | 2.69 | 0.95 | | 0.0 |
| H04 | 2.86 | 2.64 | | 0.0 |
| H05 | 2.36 | 0.24 | | 0.0 |
| H06 | 2.74 | 0.79 | | 0.0 |
| H07 | 1.99 | 1.86 | | 0.0 |
| H08 | 1.99 | 0.55 | | 0.0 |
| H09 | 2.45 | 0.71 | | 0.0 |
| H10 | 2.16 | 1.10 | | 0.0 |
| H11 | 2.05 | 2.07 | | 0.0 |
| H12 | 1.76 | 0.58 | | 0.0 |
| H13 | 2.08 | 1.38 | | 0.0 |
| H14 | 2.14 | 1.50 | | 0.0 |
| H15 | 2.77 | 0.49 | | 0.0 |
| H16 | 2.56 | 1.80 | | 0.0 |
| H17 | 2.10 | 1.30 | | 0.0 |
| H18 | 2.56 | 1.30 | | 0.0 |
| H19 | 2.49 | 0.90 | | 0.0 |
| H20 | 2.22 | 5.60 | | 0.0 |
| H21 | 2.39 | 3.00 | | 0.0 |
| H22 | 2.84 | 2.50 | | 0.0 |
| H23 | 2.46 | 0.70 | | 0.0 |
| H24 | 2.04 | 1.60 | | 0.0 |
| H25 | 2.17 | 1.60 | | 0.0 |
| H26 | 1.86 | 5.30 | 13.7 | 0.0 |
| H27 | 2.10 | 3.20 | 7.1 | 0.0 |
| H28 | 2.66 | 6.30 | 34.4 | 0.0 |
| H29 | 2.88 | 5.80 | 28.6 | 0.0 |
| H30 | 2.89 | 5.30 | 9.3 | 0.0 |
| H31 | 3.13 | 4.10 | 18.6 | 0.0 |
| H32 | 2.18 | 2.60 | 9.9 | 0.0 |
| H33 | 2.55 | 2.40 | 5.3 | 0.0 |
| H34 | 1.62 | 2.50 | 3.0 | 0.0 |
| H35 | 1.55 | 0.60 | 1.0 | 0.0 |
| H36 | 2.92 | 2.00 | 3.1 | 0.0 |
| H37 | 2.44 | 2.50 | 2.1 | 0.0 |
| H38 | 2.66 | 1.50 | 1.6 | 0.0 |
| H39 | 2.23 | 1.60 | 2.4 | 0.0 |
| H40 | 2.69 | 1.50 | 1.5 | 0.0 |
| H41 | 2.50 | 1.90 | 3.9 | 0.0 |
| H42 | 2.90 | 0.90 | 1.0 | 0.0 |
| H43 | 2.11 | 0.80 | 6.0 | 0.0 |

| | | | | |
|---|---|---|---|---|
| H44 | 2.36 | 1.60 | 1.6 | 0.0 |
| H45 | 2.90 | 4.90 | 5.6 | 0.0 |
| H46 | 2.01 | 3.30 | 3.4 | 0.0 |
| H47 | 3.02 | 3.60 | 27.5 | 0.0 |

| Non-splenectomized HbE/β-thal Subject | HbNO production at 30 sec (nmol/g heme/sec) | Baseline Plt P-selectin (%) | 1 μM ADP-induced Plt P-selectin (%) | HbE (%) |
|---|---|---|---|---|
| NSP01 | 1.89 | 7.54 | N/A | 44.6 |
| NSP02 | 1.40 | 5.12 | | 38.0 |
| NSP03 | 1.59 | 3.43 | | 65.7 |
| NSP04 | 2.42 | 5.34 | | 47.2 |
| NSP05 | 2.03 | 1.31 | | 42.4 |
| NSP06 | 2.24 | 1.99 | | 23.9 |
| NSP07 | 2.29 | 6.72 | | 46.3 |
| NSP08 | 1.60 | 0.92 | | 71.3 |
| NSP09 | 2.26 | 1.36 | | 59.9 |
| NSP10 | 2.67 | 1.38 | | 32.6 |
| NSP11 | 2.06 | 2.59 | | 62.8 |
| NSP12 | 1.69 | 1.86 | | 48.2 |
| NSP13 | 1.58 | 0.62 | | 35.0 |
| NSP14 | 1.70 | 2.50 | | 25.0 |
| NSP15 | 1.58 | 0.80 | | 46.0 |
| NSP16 | 0.45 | 4.70 | | 58.0 |
| NSP17 | 0.45 | 6.40 | | 49.6 |
| NSP18 | 1.35 | 9.90 | | 46.7 |
| NSP19 | 2.80 | 4.20 | | 42.1 |
| NSP20 | 1.97 | 2.00 | | 61.0 |
| NSP21 | 2.47 | 1.40 | | 42.1 |
| NSP22 | 2.36 | 1.20 | | 53.1 |
| NSP23 | 1.97 | 1.90 | | 54.6 |
| NSP24 | 0.80 | 3.20 | | 75.8 |
| NSP25 | 1.33 | 4.40 | | 58.1 |
| NSP26 | 1.27 | 9.20 | | 47.4 |
| NSP27 | 0.81 | 5.20 | | 60.2 |
| NSP28 | 0.95 | 6.00 | | 53.9 |
| NSP29 | 0.70 | 1.70 | | 46.6 |
| NSP30 | 1.37 | 8.80 | 21.5 | 41.8 |
| NSP31 | 0.39 | 9.80 | 54.1 | 56.2 |
| NSP32 | 1.74 | 3.60 | 32.1 | 56.1 |
| NSP33 | 1.10 | 7.30 | 10.3 | 63.3 |
| NSP34 | 0.81 | 4.10 | 7.0 | 56.2 |
| NSP35 | 0.98 | 7.20 | 39.0 | 58.4 |
| NSP36 | 0.84 | 7.20 | 8.8 | 39.5 |

| | | | | |
|---|---|---|---|---|
| NSP37 | 2.16 | 3.80 | 12.6 | 15.4 |
| NSP38 | 0.78 | 10.50 | 5.0 | 51.6 |
| NSP39 | 1.54 | 5.30 | 37.0 | 67.0 |
| NSP40 | 1.49 | 3.60 | 2.9 | 43.3 |
| NSP41 | 0.86 | 4.30 | 3.5 | 42.1 |
| NSP42 | 0.53 | 4.80 | 3.0 | 57.5 |
| NSP43 | 2.36 | 5.60 | 4.2 | 23.6 |
| NSP44 | 0.88 | 2.70 | 11.8 | 45.8 |
| NSP45 | 2.28 | 5.10 | 23.0 | 57.6 |
| NSP46 | 1.38 | 8.20 | 29.7 | 70.5 |
| NSP47 | 1.18 | 15.50 | 43.8 | 42.8 |
| NSP48 | 2.09 | 8.20 | 29.6 | 41.5 |
| NSP49 | 0.74 | 9.60 | 8.6 | 72.8 |
| NSP50 | 1.48 | 9.40 | 28.3 | 53.8 |
| NSP51 | 1.01 | 1.20 | 11.3 | 48.2 |
| NSP52 | 1.64 | 9.60 | 25.4 | 64.4 |
| NSP53 | 1.31 | 10.20 | 4.8 | 71.2 |
| NSP54 | 1.11 | 0.40 | 19.3 | 79.4 |
| NSP55 | 2.05 | 5.90 | 26.7 | 45.0 |
| NSP56 | 0.58 | 5.00 | 35.1 | 78.4 |
| NSP57 | 1.18 | 19.80 | 65.1 | 63.8 |
| NSP58 | 1.86 | 7.40 | 15.9 | 38.8 |

| Splenectomized HbE/β-thal Subject | HbNO production at 30 sec (nmol/g heme/sec) | Baseline P-selectin (%) | HbE (%) | HbF (%) |
|---|---|---|---|---|
| SP01 | 1.77 | 12.19 | 17.8 | 27.9 |
| SP02 | 0.94 | 8.19 | 71.3 | 23.4 |
| SP03 | 1.45 | 11.05 | 16.4 | 21.3 |
| SP04 | 1.81 | 3.34 | 22.9 | 17.5 |
| SP05 | 1.46 | 12.53 | 19.5 | 3.4 |
| SP06 | 1.33 | 4.89 | 65.0 | 24.3 |
| SP07 | 1.33 | 5.25 | 16.1 | 38.4 |
| SP08 | 1.85 | 9.49 | 19.0 | 60.6 |
| SP09 | 1.71 | 16.36 | 31.1 | 26.2 |
| SP10 | 1.04 | 21.73 | 48.9 | 46.5 |
| SP11 | 1.86 | 10.87 | 12.5 | 15.5 |
| SP12 | 1.61 | 6.53 | 30.8 | 46.2 |
| SP13 | 1.99 | 3.44 | 38.9 | 22.3 |
| SP14 | 1.29 | 15.55 | 36.4 | 22.9 |
| SP15 | 1.91 | 17.60 | *33.5* | 61.7 |
| SP16 | 1.47 | 23.11 | 43.2 | 26.2 |
| SP17 | 1.56 | 10.52 | 11.5 | 19.4 |
| SP18 | 1.85 | 6.36 | 22.0 | 69.1 |
| SP19 | 0.83 | 20.08 | 58.1 | 36.7 |
| SP20 | 1.22 | 27.86 | 48.50 | 48.10 |

| | | | | |
|---|---|---|---|---|
| SP21 | 1.35 | 10.63 | 60.50 | 32.60 |
| SP22 | 1.30 | 15.30 | 29.40 | 23.80 |
| SP23 | 1.66 | 2.99 | 31.1 | 13.1 |

| HbA (%) |
| --- |
| 99.6 |
| 99.9 |
| 99.6 |
| 99.8 |
| 99.8 |
| 99.8 |
| 99.8 |
| 99.9 |
| 99.4 |
| 99.6 |
| 99.4 |
| 98.8 |
| 99.8 |
| 99.7 |
| 99.6 |
| 99.6 |
| 99.9 |
| 99.6 |
| 99.8 |
| 99.7 |
| 99.8 |
| 99.8 |
| 99.6 |
| 99.4 |
| 99.6 |
| 99.4 |
| 99.3 |
| 99.6 |
| 99.6 |
| 99.9 |
| 99.6 |
| 99.8 |
| 99.7 |
| 99.8 |
| 99.8 |
| 99.6 |
| 99.4 |
| 99.6 |
| 99.4 |
| 98.9 |
| 99.9 |
| 99.7 |
| 99.6 |

| |
|---|
| 99.5 |
| 99.6 |
| 99.4 |
| 98.9 |

| HbF (%) | HbA (%) |
|---|---|
| 45.2 | 10.2 |
| 53.6 | 8.4 |
| 17.7 | 16.6 |
| 41.4 | 11.4 |
| 47.6 | 10.0 |
| 10.1 | 66.0 |
| 43.0 | 10.7 |
| 3.4 | 25.3 |
| 27.3 | 12.8 |
| 25.3 | 42.1 |
| 20.7 | 16.5 |
| 31.1 | 20.7 |
| 56.9 | 8.1 |
| 51.6 | 23.4 |
| 46.2 | 7.8 |
| 33.1 | 8.9 |
| 44.5 | 5.9 |
| 12.3 | 41.0 |
| 50.1 | 7.8 |
| 18.9 | 20.1 |
| 53.1 | 4.8 |
| 23.1 | 23.8 |
| 39.2 | 6.2 |
| 3.0 | 21.2 |
| 32.4 | 9.5 |
| 46.1 | 6.5 |
| 31.7 | 8.1 |
| 36.1 | 10.0 |
| 47.9 | 5.5 |
| 23.4 | 34.8 |
| 39.3 | 4.5 |
| 39.9 | 4.0 |
| 31.3 | 5.4 |
| 39.2 | 4.6 |
| 38.0 | 3.6 |
| 59.4 | 1.1 |

| | |
|---|---|
| 10.5 | 74.1 |
| 44.6 | 3.8 |
| 28.7 | 4.3 |
| 10.9 | 45.8 |
| 41.1 | 16.8 |
| 32.1 | 10.4 |
| 10.6 | 65.8 |
| 50.4 | 3.8 |
| 33.8 | 8.6 |
| 17.6 | 11.9 |
| 54.4 | 2.8 |
| 55.7 | 2.8 |
| 21.0 | 6.2 |
| 42.7 | 3.5 |
| 48.8 | 3.0 |
| 31.2 | 4.4 |
| 24.0 | 4.8 |
| 15.0 | 5.6 |
| 52.3 | 2.7 |
| 11.5 | 10.1 |
| 27.4 | 8.8 |
| 37.8 | 23.4 |

| HbA (%) | TRV (m/s) |
|---|---|
| 54.3 | 2.48 |
| 5.3 | 3.46 |
| 62.3 | 2.52 |
| 59.6 | 2.62 |
| 77.1 | 3.69 |
| 10.7 | 2.43 |
| 45.5 | 2.95 |
| 20.4 | 2.14 |
| 42.7 | 2.54 |
| 4.6 | 3.23 |
| 72 | 2.88 |
| 23 | 2.23 |
| 38.8 | 2.71 |
| 40.7 | 2.65 |
| 4.8 | 2.47 |
| 30.6 | 3.27 |
| 69.1 | 3.04 |
| 8.9 | 2.25 |
| 5.2 | 3.15 |
| 3.40 | 3.43 |

| | |
|---|---|
| 6.90 | 4.30 |
| 46.80 | 2.30 |
| 55.8 | 3.19 |
