# Supplementary material for: Decreased nitrite reductase activity of deoxyhemoglobin correlates with platelet activation in hemoglobin E/ß-thalassemia subjects
Source: PLoS One. 2018 Sep 20;13(9):e0203955. doi: 10.1371/journal.pone.0203955 (PMC6147434; doi:10.1371/journal.pone.0203955)
Supplement: S4 Table — Data of in vitro experiments showing the effects of deoxygenated erythrocytes from normal and HbE/ß-thal on P-selectin expression on platelets. (PDF) [file pone.0203955.s004.pdf]

| | % Inhibition of platelet P-selectin expression | | | |
|---|---|---|---|---|
| Healthy Subjects | Untreated | Deoxygenation | Deoxygenation + 20 μM ODQ | Deoxygenation + 200 μM c-PTIO |
| H14 | 11.28 | 15.52 | N/A | N/A |
| H15 | 13.00 | 24.00 | | |
| H16 | 8.20 | 17.88 | 13.05 | |
| H17 | 12.24 | 31.25 | 11.33 | |
| H18 | 8.66 | 28.96 | 12.63 | |
| H19 | 3.79 | 14.99 | 5.42 | |
| H20 | 10.94 | 22.68 | 1.47 | 11.07 |
| H21 | 15.07 | 25.31 | 0.49 | 10.09 |
| H22 | 15.98 | 18.85 | 1.40 | 4.06 |
| H23 | 11.29 | 31.41 | 7.72 | 11.06 |
| H24 | 9.65 | 19.87 | 8.99 | 5.52 |

| | % Inhibition of platelet P-selectin expression | | | |
|---|---|---|---|---|
| HbE/β-thal Subjects | Untreated | Deoxygenation | Deoxygenation + 20 μM ODQ | Deoxygenation + 200 μM c-PTIO |
| NSP20 | 10.48 | 11.09 | N/A | N/A |
| NSP21 | 5.80 | 8.00 | | |
| NSP22 | 9.57 | 12.96 | | |
| NSP23 | 7.00 | 13.18 | 13.42 | |
| NSP24 | 7.14 | 11.66 | 3.43 | 3.97 |
| NSP25 | 3.97 | 5.27 | 3.16 | 0.76 |
| NSP26 | 10.24 | 11.19 | 3.25 | 0.70 |
| NSP27 | 15.04 | 13.50 | 5.77 | 9.47 |
| NSP28 | 11.19 | 10.24 | 3.26 | 0.71 |
